# ^13^C-Metabolic flux analysis detected a hyperoxemia-induced reduction of tricarboxylic acid cycle metabolism in granulocytes during two models of porcine acute subdural hematoma and hemorrhagic shock

**DOI:** 10.3389/fimmu.2023.1319986

**Published:** 2024-01-09

**Authors:** Eva-Maria Wolfschmitt, Josef Albert Vogt, Melanie Hogg, Ulrich Wachter, Nicole Stadler, Thomas Kapapa, Thomas Datzmann, David Alexander Christian Messerer, Andrea Hoffmann, Michael Gröger, Franziska Münz, René Mathieu, Simon Mayer, Tamara Merz, Pierre Asfar, Enrico Calzia, Peter Radermacher, Fabian Zink

**Affiliations:** ^1^ Institute for Anesthesiological Pathophysiology and Process Engineering, University Hospital Ulm, Ulm, Germany; ^2^ Clinic for Neurosurgery, University Hospital Ulm, Ulm, Germany; ^3^ Clinic for Anesthesia and Intensive Care, University Hospital Ulm, Ulm, Germany; ^4^ Institute for Transfusion Medicine, University Hospital Ulm, Ulm, Germany; ^5^ Clinic for Neurosurgery, Bundeswehrkrankenhaus, Ulm, Germany; ^6^ Département de Médecine Intensive – Réanimation et Médecine Hyperbare, Centre Hospitalier Universitaire, Angers, France

**Keywords:** Bayesian modeling, glucose metabolism, glutamine utilization, hyperoxia, immunometabolism, mass isotopomer distribution, peripheral blood mononuclear cells, reactive oxygen species

## Abstract

**Introduction:**

Supplementation with increased inspired oxygen fractions has been suggested to alleviate the harmful effects of tissue hypoxia during hemorrhagic shock (HS) and traumatic brain injury. However, the utility of therapeutic hyperoxia in critical care is disputed to this day as controversial evidence is available regarding its efficacy. Furthermore, in contrast to its hypoxic counterpart, the effect of hyperoxia on the metabolism of circulating immune cells remains ambiguous. Both stimulating and detrimental effects are possible; the former by providing necessary oxygen supply, the latter by generation of excessive amounts of reactive oxygen species (ROS). To uncover the potential impact of increased oxygen fractions on circulating immune cells during intensive care, we have performed a ^13^C-metabolic flux analysis (MFA) on PBMCs and granulocytes isolated from two long-term, resuscitated models of combined acute subdural hematoma (ASDH) and HS in pigs with and without cardiovascular comorbidity.

**Methods:**

Swine underwent resuscitation after 2 h of ASDH and HS up to a maximum of 48 h after HS. Animals received normoxemia (P_a_O_2_ = 80 – 120 mmHg) or targeted hyperoxemia (P_a_O_2_ = 200 – 250 mmHg for 24 h after treatment initiation, thereafter P_a_O_2_ as in the control group). Blood was drawn at time points T1 = after instrumentation, T2 = 24 h post ASDH and HS, and T3 = 48 h post ASDH and HS. PBMCs and granulocytes were isolated from whole blood to perform electron spin resonance spectroscopy, high resolution respirometry and ^13^C-MFA. For the latter, we utilized a parallel tracer approach with 1,2-^13^C_2_ glucose, U-^13^C glucose, and U-^13^C glutamine, which covered essential pathways of glucose and glutamine metabolism and supplied redundant data for robust Bayesian estimation. Gas chromatography-mass spectrometry further provided multiple fragments of metabolites which yielded additional labeling information. We obtained precise estimations of the fluxes, their joint credibility intervals, and their relations, and characterized common metabolic patterns with principal component analysis (PCA).

**Results:**

^13^C-MFA indicated a hyperoxia-mediated reduction in tricarboxylic acid (TCA) cycle activity in circulating granulocytes which encompassed fluxes of glutamine uptake, TCA cycle, and oxaloacetate/aspartate supply for biosynthetic processes. We further detected elevated superoxide levels in the swine strain characterized by a hypercholesterolemic phenotype. PCA revealed cell type-specific behavioral patterns of metabolic adaptation in response to ASDH and HS that acted irrespective of swine strains or treatment group.

**Conclusion:**

In a model of resuscitated porcine ASDH and HS, we saw that ventilation with increased inspiratory O_2_ concentrations (P_a_O_2_ = 200 – 250 mmHg for 24 h after treatment initiation) did not impact mitochondrial respiration of PBMCs or granulocytes. However, Bayesian ^13^C-MFA results indicated a reduction in TCA cycle activity in granulocytes compared to cells exposed to normoxemia in the same time period. This change in metabolism did not seem to affect granulocytes’ ability to perform phagocytosis or produce superoxide radicals.

## 1 Introduction

Hemorrhage and traumatic brain injury (TBI) are the major determinants of outcome after severe physical injury. Hemorrhage results in tissue oxygen debt (1) due to the blood loss-related reduction of circulating blood volume and O_2_ transport capacity. In patients with TBI, it is well established that ischemic or hypoxic tissue regions can persist despite adequate systemic resuscitation measures (2). Consequently, increasing the inspired O_2_ concentration is often used during the acute management of TBI and hemorrhagic shock (HS) to alleviate harmful effects of hypoxia like hypoxia-induced inflammation (3). Theoretically, hyperoxemia could be especially beneficial for patients with atherosclerosis or coronary artery disease suffering from inherent perfusion impairment (4). In fact, we had demonstrated that therapeutic hyperoxia during the early phase of resuscitation attenuated heart and kidney injury in swine with coronary artery disease (5). Similar positive effects could manifest for TBI patients by counteracting TBI-induced vasodilation and brain edema due to allowing the reduction of intracranial pressure (ICP) while maintaining tissue oxygen supply (6). In this regard, we had recently demonstrated that targeted hyperoxemia improved neurological function in a porcine model of combined acute subdural hematoma (ASDH) and HS (7, 8).

However, therapeutic hyperoxia remains a contentious topic in critical care. Hyperoxemia causes increased reactive oxygen species (ROS) generation (6, 9), especially in the context of ischemia reperfusion injury, e.g. during resuscitation from HS. Both oxygen availability and physical injuries are known factors that can interact with immune cell behavior and metabolism and potentially affect their effector functions (10, 11). Furthermore, hyperoxemia has been associated with increased oxidative stress in a broad variety of cells (12–14) including platelets and leukocytes. It is theorized that this increase in oxidative stress could potentially impact the immune and coagulatory functions of these cell subsets (14, 15).

Immunometabolism explores how changes in the metabolism of immune cells direct and guide their function (16, 17). These metabolic alterations can be elucidated by metabolic flux analysis (MFA), a technique for calculating production, consumption, and transformation rates within a biological system (18, 19). MFA most commonly relies on balancing fluxes within a stoichiometric network and employing nonlinear regressions. We have adapted MFA to a Bayesian approach, which is more computationally intensive than the conservative method, but in turn yields full probability distributions of fluxes, their joint credibility intervals, and their correlations (20). With a parallel tracer approach of 1,2-^13^C_2_ glucose, ^13^C_6_ glucose, and ^13^C_5_ glutamine we were able to cover major glycolytic and glutamine-fueled pathways like glycolysis, the pentose phosphate pathway (PPP), and the tricarboxylic acid (TCA) cycle.

In the present study, we utilized high resolution respirometry and ^13^C-MFA to investigate whether shock and targeted hyperoxemia impact the immunometabolism of circulating immune cells, specifically peripheral blood mononuclear cells (PBMCs) and granulocytes, in healthy and atherosclerotic pigs. We further investigated immune cell function by analyzing their capacity to perform phagocytosis and produce ROS with flow cytometry and electron spin resonance (ESR) spectroscopy, respectively. As hyperoxia could either have beneficial or ROS-mediated detrimental effects on immune cells, we placed special focus on the interplay between metabolic pathways and superoxide (O_2_^•−^) production. For this reason, we analyzed systematic changes in metabolism and function with a principal component analysis (PCA), which allowed capturing both cell type-specific and time-dependent metabolic plasticity of circulating immune cells during combined porcine ASDH and HS, and resuscitation. With these methods, we explored cell type-specific immunometabolic patterns of adaptation with the potential effects of *i)* exposure to hyperoxia, *ii)* cardiovascular co-morbidity, and *iii)* the response to combined ASDH and HS, and resuscitation *per se*.

## 2 Methods

### 2.1 Animals, preparation, and sample origin

**Figure 1:**
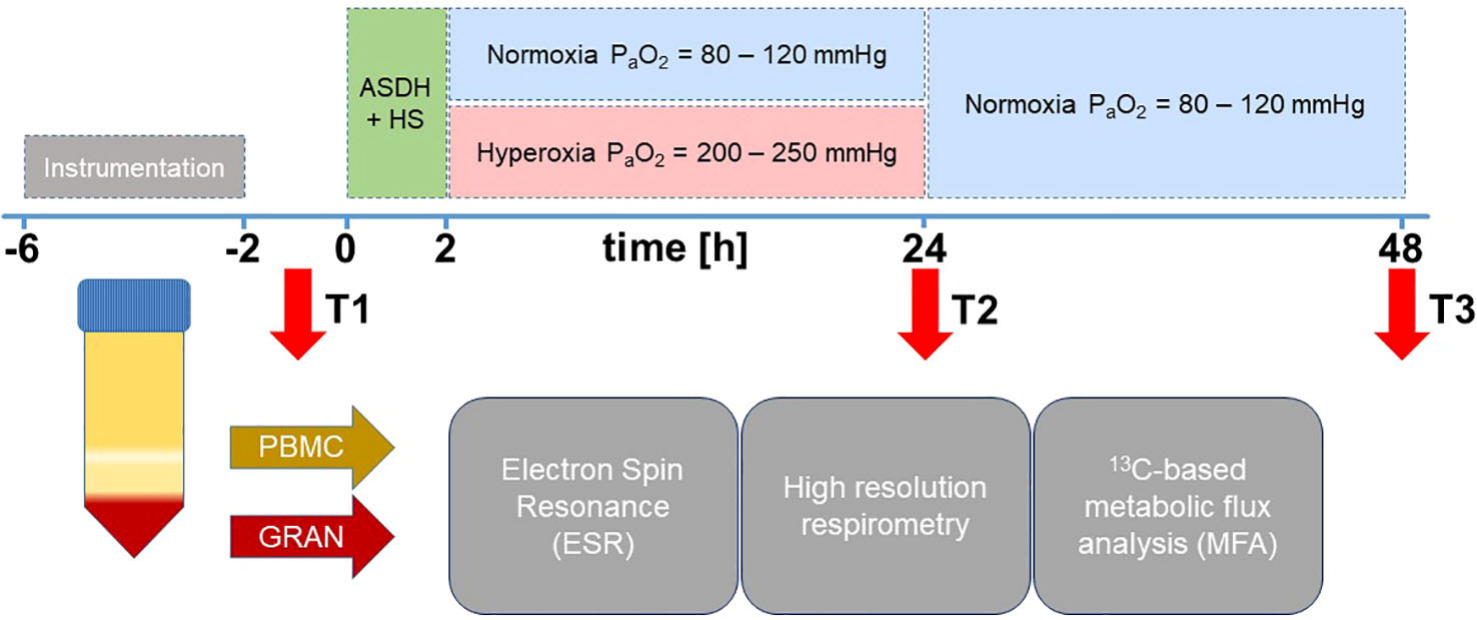
Experimental setup. After instrumentation, pigs were subjected to ASDH and HS by injection of 0.1 mL/kg autologous blood into the subdural space and passive removal of 30% of the calculated blood volume. After 2 h of ASDH and shock, swine underwent resuscitation comprising retransfusion of shed blood, fluid resuscitation and i.v. NoA titration to maintain MAP at pre-shock levels and CPP ≥ 75 mmHg. Intensive care was maintained up to a maximum of 48 h. Animals were randomly assigned to control (normoxemia, target P_a_O_2_ = 80 – 120 mmHg) or targeted hyperoxemia (target P_a_O_2_ = 200 – 250 mmHg for 24 h after treatment initiation. Thereafter, target P_a_O_2_ as in the control group). Resuscitation was maintained to a maximum of 48 h after shock before experiment termination. Blood was drawn at time points T1 = after instrumentation, T2 = 24 h post ASDH and HS, and T3 = 48 h post ASDH and HS. PBMCs and granulocytes were isolated from whole blood to perform ESR for superoxide quantification, high resolution respirometry and ^13^C-MFA.

Experiments were performed according to the guidelines of the National Institute of Health on the Use of Laboratory Animals and the European Union “Directive 2010/63 EU on the protection of animals used for scientific purposes” after approval from the local Animal Care Committee of Ulm University and the Federal Authorities for Animal Research (Regierungspräsidium Tübingen, Germany, Reg.-Nr. 1316). This study is an exploratory *post hoc* analysis of blood samples obtained during two long-term studies of combined ASDH and HS investigating swine with or without coronary artery disease over a period of three days (7, 8). The experimental procedures were identical for both studies except for the utilization of the two different swine strains (7, 8). Data of studies pertaining to survival, hemodynamics, gas exchange, acid-base state, brain perfusion, oxygenation, and neurologic outcome are reported in the respective publication. Briefly, animals were instrumented and ASDH was induced by injection of 0.1 mL/kg autologous blood into the subdural space. HS was initiated by passive removal of 30% of the animals’ calculated blood volume under maintenance of a cerebral perfusion pressure (CPP, difference between mean arterial pressure (MAP) and ICP) ≥ 50 mmHg. For resuscitation, the shed blood was returned 2 h after induction of HS and noradrenaline (NoA) was continuously administered intravenously as required to restore the MAP to pre-shock levels and maintain CPP ≥ 75 mmHg. Animals were randomly assigned to control (normoxemia (NormOx), target arterial partial pressure of oxygen (P_a_O_2_) = 80 – 120 mmHg) or targeted hyperoxemia (HyperOx, target P_a_O_2_ = 200 – 250 mmHg for 24 h after treatment initiation, thereafter target P_a_O_2_ as in the control group). Resuscitation was maintained to a maximum of 48 h after shock before experiment termination. The experimental protocol is visualized in **Figure 1**.

The first data set originated from a study that investigated Familial hypercholesterolemia-Bretoncelles Meishan (FBM) pigs. It comprised 14 adult pigs of either sex (6 females, 8 castrated males) with a median body weight of 63 kg (interquartile range (IQR) 56;71) and a median age of 38 months (IQR 36;41). This strain is characterized by a homozygous R84C mutation in the low-density lipoprotein receptor, making them susceptible to atherosclerosis and coronary artery disease. Animals were fed a cholesterol-enriched diet for at least 9 months to encourage atherosclerotic progression.

The second analyzed data set included 14 adult pigs of the Bretoncelles-Meishan-Willebrand (BMW) strain with a median body weight of 75 kg (IQR 73;76) and an age of 16 months (IQR 15;18) of both sexes (4 females and 10 castrated males). As pigs are typically hypercoagulatory (21) and this strain presents with reduced activity of the von Willebrand factor, the resulting reduced coagulatory phenotype resembles that of humans (22). In contrast to the coronary artery disease present in the FBM strain, BMW are cardiovascularly healthy (7, 8, 23, 24).

### 2.2 Cell isolation from whole blood

We isolated PBMCs and granulocytes from whole blood samples to detect changes in immunometabolism in response to combined ASDH and HS and hyperoxemia as previously described (20, 25). Blood was drawn at three different time points over the course of the experiment: T1: after (neuro)surgical instrumentation following a stabilization period; T2: 24 h after ASDH and HS, i.e. at the end of the exposure to targeted hyperoxia; T3: 48 h after ASDH and HS, i.e. at the end of the intensive care period. The precise timeline is depicted in **Figure 1**. Approximately 125 mL of arterial blood were drawn in lithium heparin monovettes (9 mL, Sarstedt, Nümbrecht, Germany) and 1:1 (v/v) diluted with PBS (without CaCl_2_, MgCl_2_) at each time point. For cell separation, the dilution was layered on top of two density gradient solutions (9 mL 1.119 and 8 mL 1.088 g/mL solution, Pancoll, PAN Biotech, Aidenbach, Germany) and centrifuged at 764 g for 20 min at room temperature (RT) with the break deactivated. This procedure yielded a PBMC top layer and a bottom layer containing red blood cells (RBCs) and granulocytes. RBC residuals were removed by osmotic lysis. A single lysis was sufficient for PBMCs, while the granulocyte-RBC layer had to be subjected to osmotic lysis three times before all RBC contamination was removed. Subsequently, cells were washed once with 1× PBS and counted in a Neubauer counting chamber.

### 2.3 High resolution respirometry

Mitochondrial respiration of isolated cells was derived from the measurement of O_2_ consumption with high resolution respirometry. We used the Oroboros-2K (Oroboros Instruments, Innsbruck, Austria) for this purpose, which is a device for simultaneous recording of the O_2_ concentration in two parallel chambers (20, 25). Briefly, chambers were calibrated with 2 mL of mitochondrial respiration medium MiR05 (composition available in **Supplementary Table 1**) adjusted to pH 7.1 with KOH and equilibrated with 21% O_2_ at 37°C. For measurement of cell respiration, we filled each chamber with 10× 10^6^ PBMCs/granulocytes suspended in MiR05. Within the chambers, cells were continuously stirred at 750 rpm. After sealing, the first derivative of the O_2_ concentration was recorded and yielded the oxygen flux (*J*O_2_), which was normalized for the cell number. The subsequent injection of substrates and inhibitors enabled the analysis of specific mitochondrial respiration functions: i) Routine respiration was recorded prior to injecting inhibitors or substrates once a stable *J*O_2_-value was achieved. ii) The LEAK-state represents the respiratory activity required to maintain a stable membrane potential in absence of ATP-turnover and was measured after addition of 2.5 μM oligomycin to block the ATP-synthase. iii) The ETS-state is defined as the maximum respiratory activity in the uncoupled state and corresponds to state 3 of Chance and Williams et al. (26). It was achieved by titration of carbonyl cyanide p-(trifluoromethoxy)-phenylhydrazone (FCCP) in 1 µM steps.

### 2.4 Quantification of superoxide anion with electron spin resonance spectroscopy

Immediately after arterial blood withdrawal at the same time points as for cell isolation, 25 µL of whole blood were mixed 1:1 (v/v) with CMH spin probe solution containing 400 µM CMH spin probe (1-hydroxy-3-methoxycarbonyl-2,2,5,5-tetramethylpyrrolidine), 25 µM deferoxamine, and 5 µM diethyldithiocarbamate solved in Krebs-HEPES-Buffer (KHB) (Noxygen, Elzach, Germany). As previously described (20, 25), the mixture was transferred to a 50 µL glass capillary, sealed, and placed in an EMXnano ESR spectrometer (Bruker, Billerica, MA, USA). After a 5 min incubation period at 37°C, the measurement was started with device settings detailed in the **Supplementary File**. O_2_^•−^ quantification was performed with eight radical standards of various CP^•^ (3-Carboxy-2,2,5,5-tetramethyl-1-pyrrolidinyloxy) concentrations solved in KHB. The blank sample (KHB mixed 1:1 with spin probe solution) was subtracted from the result for final values.

For the determination of O_2_^•−^ production by immune cells, 25 µL of cell suspension (2.5× 10^6^ cells/mL RPMI 1640 medium (glucose 1.8 mg/mL, glutamine 0.6 mg/mL, NaHCO_3_ 100 µg/mL)) were mixed 1:1 (v/v) with CMH spin probe solution directly prior to the measurement. Samples were measured 8 times over a 30 min interval to calculate O_2_^•−^ production rates. All other settings were analogous to whole blood radical determination. The blank sample (RPMI 1640 mixed 1:1 with spin probe solution) was subtracted from the result for final values. We used the Xenon_nano software (version 1.3; Bruker BioSpin GmbH, Rheinstetten, Germany) and Microsoft Excel for data analysis.

### 2.5 Phagocytosis assay

Purified granulocytes were resuspended in fluorescence-activated cell sorting (FACS) buffer + 10% pig serum (Bio-Rad Laboratories, Hercules, CA, USA) and kept on ice before staining. Cells were used within 3 h from blood withdrawal and 1 h from purification. Granulocytes were treated according to manufacturer’s instructions of the pHrodo™ Bioparticles Phagocytosis Kit for Flow Cytometry (Thermo Fisher Scientific, Waltham, MA, USA). Briefly, 10× 10^6^ granulocytes, were initially blocked with 100% pig serum and incubated on ice for 10 min. 1× 10^6^ cells per sample were transferred into 100 µL RPMI-medium and kept on ice for further processing. Negative control on ice (Nci) and Negative control at 37°C (Nc37), each containing cells only, were incubated at 4°C (Nci) or 37°C (Nc37) for 20 min. Positive Control on ice (Pci), Positive control at 37°C (Pc37) and samples were incubated with 20 µL of 1 mg/mL pHrodo^®^ *E.coli* BioParticles^®^ conjugate at 37°C for 10 min and 20 min (Pci and Pc37), respectively. Phagocytosis was stopped by transferring the samples onto ice and washing with washing buffer. The samples were subsequently resuspended in FACS buffer containing 10% pig serum for incubation with porcine-specific antibodies against granulocyte markers (2B2, 6D10; Bio-Rad Laboratories, Hercules, CA, USA). The samples were transferred to FACS buffer without pig serum and kept on ice until further analysis with a Beckman Coulter CytoFLEX Flow Cytometer (Beckman Coulter Life Sciences, Brea, CA, USA).

Granulocytes were gated according to their forward (FSC) and side scatter (SSC) characteristics in conjunction with porcine granulocyte specific antibody staining (2B2^+^, 6D10^+^). Fluorescence spillover was corrected by compensation using single stained porcine specific antibodies and pHrodo™ signal. Gating approaches included post-density gradient separation, live/dead cell distinction (PI-staining), exclusion of doublet signals (FSC-H vs. FSC-A blot), and pHrodo-positive granulocyte populations (6D10^+^, 2B2^+^, pHrodo^+^). A total of 20,000 events were acquired for each sample. The phagocytic activity of each population was foremost measured by the median fluorescent intensity (MFI) of the pHrodo signal (PE-channel) and subsequently normalized (27). Briefly, MFI scores of Pc37 were divided by the MFI of their dedicated sample with thermally inhibited phagocytosis at 4°C (Pci), concluding with normalized MFI (nMFI) as describing dimension. Thus, phagocytic activity was calculated using Equation 1:


(1) $$\text{nMFI}=\frac{MFI\;sample\;incubated\;at\;37{}^{\circ}C(\text{Pc}37)}{MFI\;inhibited\;phagocytosis(\text{Pci})}$$

As the fluorogenic pHrodo dye increases fluorescence as its surroundings become more acidic, PE signal intensity directly correlated with internalized particles. For statistical validation, positive control at 37°C (Pc37) samples were tested as triplicates, all other samples were measured as singular values. All samples were analyzed with CytExpert 2.4 software (Beckman Coulter Life Sciences, Brea, CA, USA).

### 2.6 *Ex vivo* ^13^C tracer experiments

This method has been previously utilized and described in detail in Wolfschmitt et al. (20). For ^13^C parallel tracer experiments, we incubated three times 5× 10^6^ cells in 1 mL RPMI supplemented with one of the following tracers each: 1,2-^13^C_2_ glucose, ^13^C_6_ glucose, and ^13^C_5_ glutamine (Cambridge Isotope Laboratories, Andover, MA, USA). Directly prior to the start of the 3-day experiment, the respective medium was supplemented with the tracers and NaHCO_3_ and pH was adjusted to 7.4 by addition of 1M HCl or NaOH. Final concentrations are specified in **Supplementary Table 2**. Cells were incubated at 37°C for 2 h for ^13^C labeling experiments. For analysis of stimulated granulocyte metabolism, 5× 10^6^ of freshly isolated cells were washed with RPMI and subsequently resuspended in 200 µL RPMI + 10% pig serum. 50 µL of pHrodo^®^ *E.coli* BioParticles^®^ conjugate were added to the cells and tubes were incubated in a water bath at 37°C for 20 min. Afterwards, cells were washed with 1 mL washing buffer and then resuspended in 1 mL RPMI supplemented with isotopic tracers. All other steps followed those of the parallel tracer approach of granulocytes/PBMCs.

After incubation for 2 h, 850 µL of supernatant were transferred to a crimp neck glass vial after spin down centrifugation at 4°C for analysis of ^13^CO_2_ production and lactate released into the medium. The vial was frozen upside down at −20°C to prevent gas from escaping until gas chromatography/mass spectrometry (GC/MS) analysis. The leftover supernatant was discarded. Pellets were frozen at −80°C after washing them once with 1× PBS and removal of all liquid.

#### 2.6.1 Quantification of ^13^CO_2_ release

The cumulative cellular ^13^CO_2_ production was calculated with enrichment analysis. We injected 25 µL 1M HCl through the septum into the thawed supernatant and shook the sample to release CO_2_ from the NaHCO_3_ buffer system into the gaseous phase. Each sample was measured 10 times with each 5 µL headspace gas injected into the GC/MS system (Agilent 6890 GC/5975B MSD, Agilent Technologies, Waldbronn, Germany). To capture both labeled and unlabeled CO_2_, we analyzed the *m/z* of 44 and 45 in the selected ion monitoring (SIM) mode. The isotopic enrichment tracer-to-tracee-ratio (TTR) was calculated for each sample according to Equation 2:


(2) TTR=(C13O2C12O2)sample−(C13O2C12O2)background

This resulted in the calculation of the ^13^CO_2_ production with Equation 3


(3) C13O2 production[nmol1×106cells]=TTR×E[μM]cell density[1×106cellsmL]

where E is the concentration of sodium bicarbonate in the RPMI medium. After analysis, the medium was frozen at −20°C until lactate measurement with GC/MS.

#### 2.6.2 Measurement of lactate released into the medium

For lactate quantification, two times 100 µL of supernatant were thawed and one of the 100 µL samples was spiked with 1 µg of internal standard (IS, corresponding to 20 µL of 50 µg/mL ^13^C_3_ sodium lactate solution). 500 µL acetonitrile were added to each replicate. After centrifugation (13000 rpm, 5 min, RT), samples were decanted into vials suited for derivatization and dried in a Savant2010 SPD 2010 SpeedVac concentrator (Thermo Scientific, Waltham, MA, USA) (45°C, 14 mTorr) for about 50 min. For derivatization of lactate, 100 µL acetonitrile and 25 µL N-(tert-butyldimethylsilyl)-N-methyltrifluoroacetamide (MTBSTFA, abcr, Karlsruhe, Germany) were added and samples were incubated at 80°C for 1 h. Standard samples comprised the following: 0.1 µg/0.2 µg/0.5 µg/0.75 µg/1 µg of sodium lactate with an additional 1 µg of IS each, samples with 1 µg of IS only, samples with blank RPMI, and samples RPMI with 1 µg of IS. Detailed equations for lactate quantification can be found in the *supplements* of Wolfschmitt et al. (20).

#### 2.6.3 Detection of intracellular metabolites

All steps of extraction were performed on ice to avoid enzyme activation. For metabolite extraction from intracellular compartments, pellets were resuspended in 100 µL of ice-cold water, sonicated for 10 min, and mixed with 500 µL of acetonitrile. After centrifugation, the clear supernatant was transferred into a GC/MS vial suitable for derivatization. In analogy to the measurement of supernatant-derived lactate, we derivatized the intracellular metabolites with MTBSTFA. Steps of derivatization are mentioned in *2.6.2*. Standards were prepared as follows: one standard series with 0.1 µg/0.2 µg/0.5 µg/0.75 µg/1 µg of lactate and a mix with the respective amount of aspartate, glutamine, and glutamate.

We performed GC/MS detection of TBDMS metabolites and corresponding suitable ion fragments (**Supplementary Table 4**) with SIM for an optimal signal to noise ratio. SIM methods, device settings, and oven programs are mentioned in the **Supplementary File**. Peak area integration was performed with our in-house program (28). Mass isotopomer distributions (MIDs) were converted into carbon mass distributions (CMDs) by deducting isotopic interferences with a correction matrix approach (20, 29). It is important to note that our definition of “labeling” includes natural ^13^C abundance, which was not corrected for.

### 2.7 Metabolic flux analysis

We have implemented a model for combined PPP/glycolysis and TCA cycle metabolism in RStan (R interface to stan, package rstan) (30). This model has been utilized in a previous form in Stifel et al. (31) and in a similar scope in our work investigating the effect of sodium thiosulfate on circulating immune cells (20).

Rstan is a sampling-based tool for Bayesian statistics. In our implementation, it draws random values for all parameters (fluxes) and calculates the corresponding theoretical CMDs for metabolites in the network according to our model description. If the theoretical CMDs come close to the actual GC/MS data, the value is considered a true value of the underlying posterior distribution and collected in a Markov chain Monte Carlo (MCMC) chain. The mathematical model of the biological network used for CMD calculations was built with the elementary metabolite unit (EMU) strategy (32). Detailed model explanations can be found in the *supplements* of Wolfschmitt et al. (20). Statistical properties like posterior mean, posterior standard deviations and credibility intervals were derived from the final MCMC chain of each parameter. Our priors were chosen to be relatively uninformative. Details of parameter bounds and prior specifications are listed in **Supplementary Tables 5** and **6**. Quantification was achieved by inclusion of ^13^CO_2_ production and lactate secretion into the medium. Briefly, ratios were determined by CMD data only with a first Rstan model and then transformed into absolute values with another stan model by accordingly scaling ratios to fit lactate release and ^13^CO_2_ production data. The parallel tracer setup of 1,2-^13^C_2_ glucose, ^13^C_6_ glucose, and ^13^C_5_ glutamine enabled improved flux determination, as all posterior parameters must apply to sets of measurements obtained from each tracer.

### 2.8 Statistical analysis

This study included samples from 14 FBM and 14 BMW pigs. Animals were randomly assigned to Hyperoxia or Normoxia treatment groups (FBM: NormOx and HyperOx; BMW: NormOx and HyperOx; each n = 7) (7, 8). Data are presented as median with IQR. Due to the nature of this project, a previous sample size determination was impossible due to the lack of preexisting data. Furthermore, original power estimations were calculated with clinical parameters as primary criteria rather than the immunometabolic analysis. Therefore, missing measurement time points for T1 and T3 due to insufficient cell yield and/or premature termination of an experiment greatly impacted our options for statistical evaluations (e.g. the use of repeated measures methods). For this reason, we present individual data points for better transparency and emphasize the exploratory nature of this study. P values should be treated with the latter in mind. Experimental data were considered to be non-parametric due to small sample sizes. We conducted the comparison between groups with Mann-Whitney U tests, while the effect of time within one group was analyzed with the Kruskal-Wallis rank sum test and a *post hoc* Dunn’s multiple comparisons test. Statistical and graphical presentation was performed with GraphPad Prism 10, version 10.0.2 (GraphPad Software Inc., La Jolla, CA, USA).

The PCA was performed with varying combinations of the following data sets: FBM PBMCs (n = 34), BMW PBMCs (n = 28), FBM granulocytes (n = 27), BMW granulocytes (n = 31), and BMW *E.coli* bioparticle-stimulated granulocytes (n = 31). All relevant fluxes were included in this analysis, as well as data from O_2_^•−^ production and mitochondrial Routine respiration (n = 22 parameters total). Data were standardized to a sample mean of zero and a unit sample SD of 1. The PCA was performed in R (version 4.3.1 (2023-06-16 ucrt)) and RStudio (version 2023.06.1) with prcomp, varimax, pracma and fviz_eig. To ensure PCA stability, we employed the jackknife method, a cross validation technique that works according to a “leave one out” principle. Briefly, it estimates a parameter for the whole data set and subsequently skips one element before recalculating the parameter as a “partial estimate” (33). This procedure was extended to correct for a directional change in the scores and loadings (“reflection”, as proposed by (34)) and was repeated until each element had been dropped once. Mean and SD of all partial estimates were then calculated. If the mean for the PCA parameter was still significant, this parameter was considered for interpretation as a significant contributor.

## 3 Results

This is an exploratory study of data obtained from two studies investigating the effects of hyperoxia during ASDH and HS in different swine strains. In the following, we will discuss the respective impact of targeted hyperoxia, swine strain, and ASDH and HS on immune cell behavior. We have analyzed each of these factors according to their influence on immunometabolism. For this reason, we took both absolute values and the differences between measurement time points into consideration. The latter indicated the progression of individual animals over time, as it excluded pre-existing particularities presented at baseline.

### 3.1 Targeted hyperoxia dampened TCA cycle activation in circulating granulocytes

**Figure 2:**
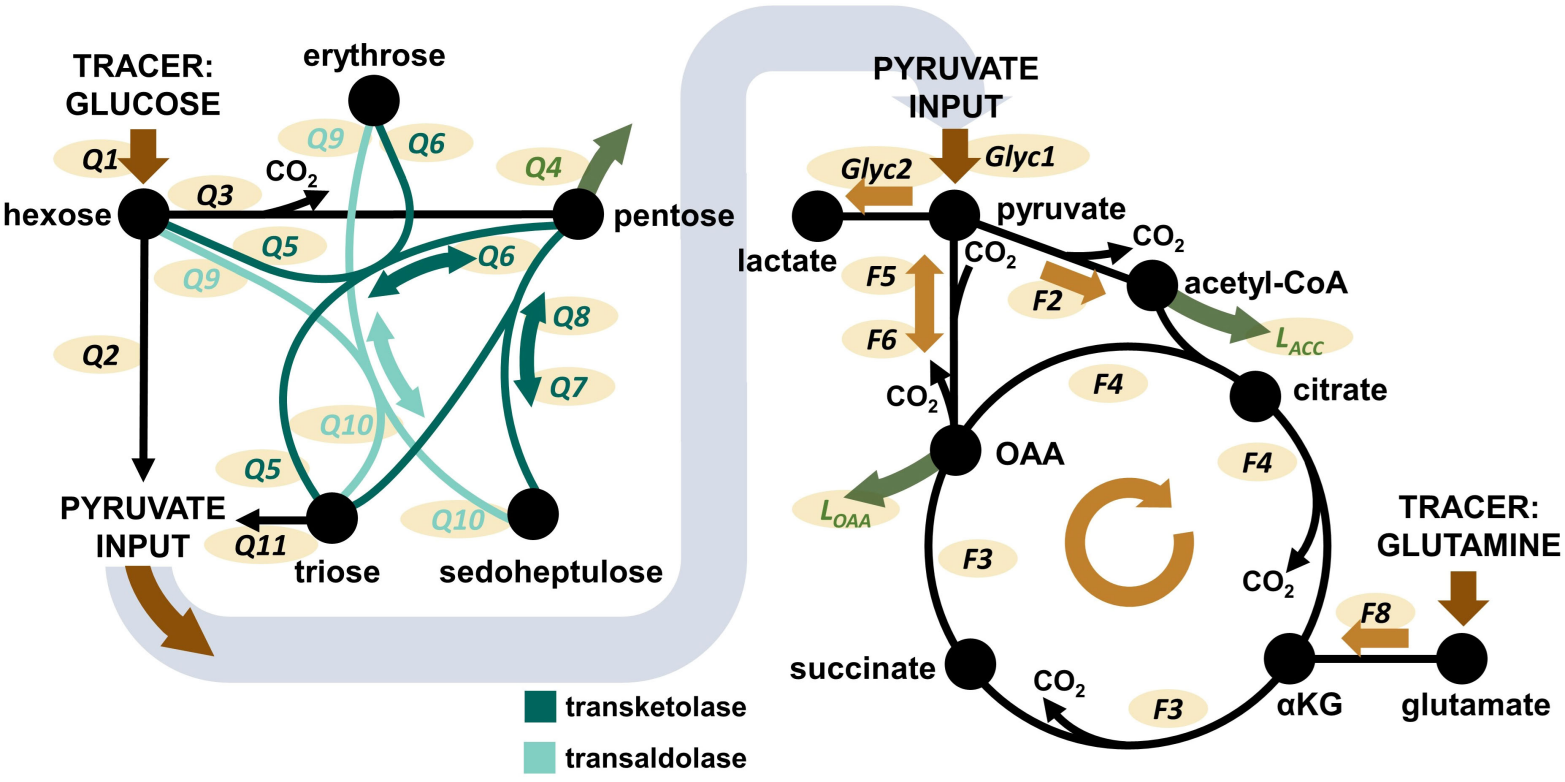
Overview over the pathways covered by the Bayesian ^13^C-MFA model. Left: PPP model. Right: TCA cycle model. Glyc: glycolytic flux. Q: flux within the PPP. F: flux within the TCA cycle. L: flux of metabolite leaving the network, e.g. for biosynthetic processes. Additional considered metabolite inputs (pyruvate, OAA, acetyl-CoA) and losses (pyruvate) are not shown in the graphic, but are presented and visualized in **Supplementary Figure 1**. The Figure is taken from Wolfschmitt et al. (20), with permission.

As a first factor we investigated the impact of increasing O_2_ availability on the metabolism of circulating immune cells with a focus on changes that persist after *ex vivo* isolation of PBMCs and granulocytes from whole blood. In this context, the metabolic network comprising glycolysis, PPP, and the TCA cycle was of particular interest (**Figure 2**). The complete metabolic model with all flux balances is provided in the **Supplementary Figure 1**. Animals had been exposed to targeted hyperoxia or control between time points T1 and T2 for a period of 24 hours. In conclusion, we observed both the absolute values of groups at T2 as well as the time-related development between those two measurement time points (i.e., the difference between values at T1 and T2 for individual animals, ΔT2-T1). As data from both swine strains were pooled for this analysis, we have indicated the respective origin of each data point in the graphs.

**Figure 3:**
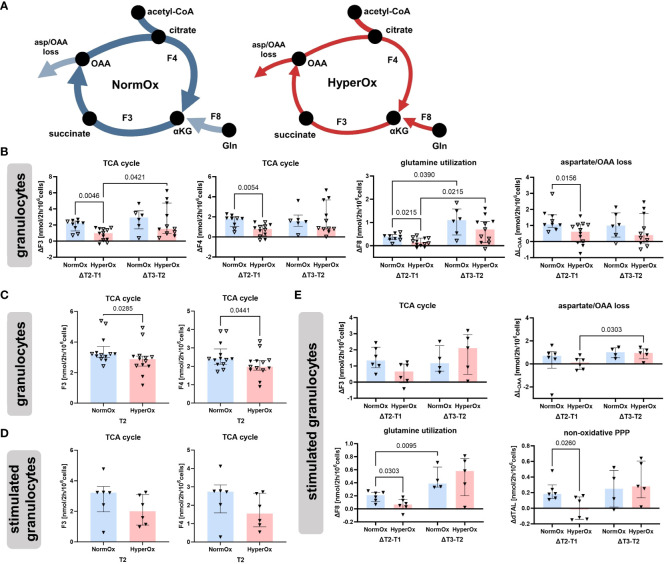
Effects of targeted hyperoxia on circulating granulocytes. Triangles with filled symbols indicate data originating from BMW animals, and empty symbols FBM data. Bars indicate the median with IQR (NormOx: blue bars, HyperOx: red bars). **(A)** The increase in TCA cycle activity from the first to the second measurement time point was reduced after cells were exposed to targeted hyperoxia. **(B)** Differences in time-related behavior of fluxes between NormOx and HyperOx groups. On the left side of each graph is the change in flux between T1 and T2, on the right side the change between T2 and T3. ΔT2-T1: NormOx (FBM n = 3, BMW n = 6), HyperOx (FBM n = 6, BMW n = 6), and ΔT3-T2: NormOx (FBM n = 2, BMW n = 4), HyperOx (FBM n = 6, BMW n = 5). **(C)** Absolute flux values of central TCA cycle fluxes at T2. Graphs including all measurement time points are available in **Supplementary Figure 2**. NormOx (FBM n = 7, BMW n = 6), HyperOx (FBM n = 7, BMW n = 6). **(D)** Absolute values of central TCA cycle fluxes at T2 in isolated granulocytes after *ex vivo* stimulation with *E.coli* bioparticles. Graphs including all measurement time points are available in **Supplementary Figure 3**. NormOx (BMW n = 6) and HyperOx (BMW n = 6). **(E)** Differences in time-related behavior of fluxes between NormOx and HyperOx groups in isolated granulocytes after stimulation with *E.coli* bioparticles. ΔT2-T1: NormOx (BMW n = 6), HyperOx (BMW n = 6), and ΔT3-T2: NormOx (BMW n = 4), HyperOx (BMW n = 4). F3: flux from α-ketoglutarate to oxaloacetate, F4: oxaloacetate to α-ketoglutarate. F8: glutamate to α-ketoglutarate. L_OAA_: flux indicating amount of oxaloacetate/aspartate leaving the investigated network. dTAL: net flux in non-oxidative PPP activity (Q9-Q10). P-values are indicated in the graphs. We performed Mann-Whitney U tests for intergroup differences, and Kruskal-Wallis rank sum tests for time-related effects.

**Figure 3** shows flux results obtained for granulocytes. The most striking difference between HyperOx and NormOx groups was seen in glutamine-fueled TCA cycle activity, as HyperOx-treated granulocytes displayed a dampened increase in TCA cycle fluxes (F3, F4), glutamine utilization (F8), and aspartate/oxaloacetate “loss” (L_OAA_) from T1 to T2 (Δ T2-T1) (**Figure 3B**). In our analysis, “losses” were defined as the flux of metabolite leaving the described metabolic network, e.g., for protein or nucleotide biosynthesis. In addition, also the absolute values for TCA cycle fluxes (F3, F4) were reduced in the HyperOx group at T2, i.e., the end of targeted hyperoxemia treatment (**Figure 3C**). In summary, the typically occurring increase in TCA cycle activity from the first to the second measurement time point was dampened under targeted hyperoxia (**Figure 3A**).

The HyperOx-mediated reduction in TCA cycle fluxes was still visible in granulocytes after *ex vivo* stimulation with *E.coli* bioparticles for both absolute (**Figure 3D**) and ΔT2-T1 values (**Figure 3E**). Interestingly, stimulated HyperOx granulocytes further displayed reduced ability to increase non-oxidative PPP (dTAL, net flux Q9-Q10) fluxes from T1 to T2 (**Figure 3E**). However, phagocytic stimulation was only performed for BMW animals, so data analysis suffered from low sample availability. This is also reflected in the rather large data spread.

**Figure 4:**
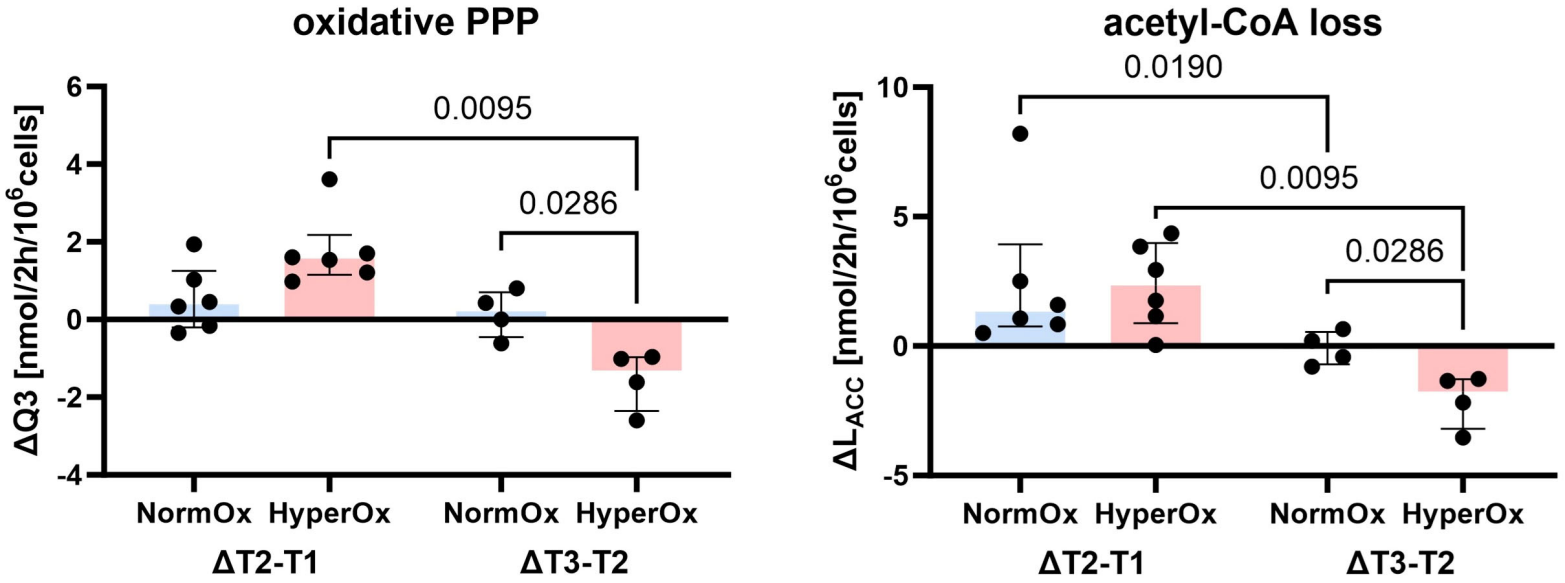
Effects of targeted hyperoxia on circulating PBMCs of BMW animals. Bars indicate the median with IQR (NormOx: blue bars, HyperOx: red bars). Fluxes with differences in time-related development between NormOx (ΔT2-T1: n = 6, ΔT3-T2: n = 4) and HyperOx (ΔT2-T1: n = 6, ΔT3-T2: n = 4) groups in isolated PBMCs from BMW animals. Q3: flux representing oxidative PPP activity. L_ACC_: flux indicating amount of acetyl-CoA leaving the investigated network. P-values are indicated in the graphs. We performed Mann-Whitney U tests for intergroup differences, and Kruskal-Wallis rank sum tests for time-related effects.

Targeted hyperoxia-mediated effects on circulating PBMCs were less pronounced. We found a hyperoxia-induced decrease in oxidative PPP utilization (Q3) from T2 to T3, while NormOx PBMCs increased Q3 in the same time frame. This decrease in ΔQ3 coincided with the same behavior in acetyl-CoA loss (L_ACC_). Both pathways are involved in lipid synthesis, the former by providing NADPH and the latter by supplying acetyl-CoA. However, this effect only applied to BMW animals (**Figure 4**). Overall, effects of targeted hyperoxia on PBMCs pertained to the readaptation to normoxia rather than immediate effects at T2 and did not apply to animals with cardiovascular co-morbidity.

Graphs depicting absolute values for all measurement time points, O_2_^•−^ production, and respiration are available in the supplements (**Supplementary Figures 2–5**).

### 3.2 Strain-specific differences in immunometabolism

**Figure 5:**
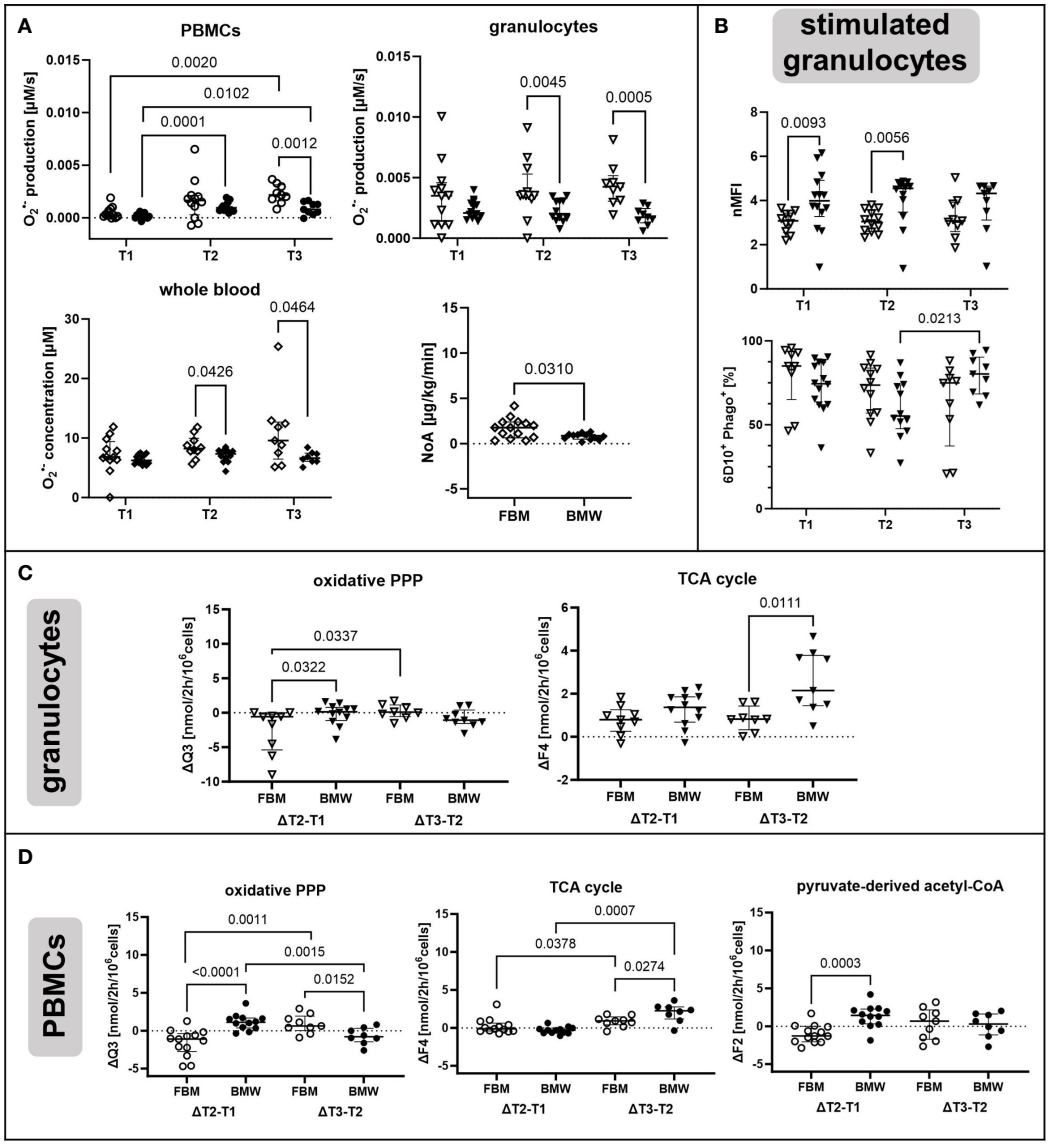
Strain-specific effects of ASDH and HS on immunometabolism. Diamonds indicate non-cell type-specific data. PBMCs are depicted as circles and granulocytes as triangles with filled symbols indicating data originating from BMW animals, and empty symbols FBM data. **(A)** O_2_^•−^ production by PBMCs and granulocytes and O_2_^•−^ concentration in whole blood as determined by ESR at the indicated measurement time points. Averaged noradrenalin (NoA) amount required during the experiment by FBM and BMW animals. PBMCs and granulocyte production: T1 (FBM n = 12, BMW n = 14), T2 (FBM n = 12, BMW n = 12), T3 (FBM n = 9, BMW n = 9). Whole blood concentration: T1 (FBM n = 12, BMW n = 14), T2 (FBM n = 10, BMW n = 12), T3 (FBM n = 9, BMW n = 8). NoA administration: FBM n = 14, BMW n = 12. **(B)** Phagocytic activity of *E.coli* bioparticle-stimulated granulocytes. Data is either presented as normalized mean fluorescence intensity (nMFI) or fraction of phagocytic granulocytes within the granulocyte subset (6D10^+^ Phago^+^). T1 (FBM n = 10, BMW n = 14), T2 (FBM n = 12, BMW n = 12), T3 (FBM n = 9, BMW n = 9). **(C)** Fluxes with differences in time-related development in granulocytes between FBM and BMW groups. On the left side of each graph is the change in flux between T1 and T2, on the right side the change between T2 and T3. ΔT2-T1: FBM n = 9, BMW n = 12, and ΔT3-T2: FBM n = 8, BMW n = 9. **(D)** Fluxes with differences in time-related development in PBMCs between FBM and BMW groups. ΔT2-T1: FBM n = 13, BMW n = 12, and ΔT3-T2: FBM n = 9, BMW n = 8. Q3: flux representing oxidative PPP activity. F2: pyruvate-derived fraction of acetyl-CoA. F4: flux from oxaloacetate to α-ketoglutarate. P-values are indicated in the graphs. We performed Mann-Whitney U tests for intergroup differences, and Kruskal-Wallis rank sum tests for time-related effects.

As a second factor, we were interested in distinct and characteristic immunometabolic patterns that can be attributed to the different phenotypes of the FBM and BMW swine strains. **Figure 5A** shows that PBMCs and granulocytes from the hypercholesterolemic FBM animals displayed increased O_2_^•−^ production levels compared to BMW animals after T1. With progressing measurement time points, this trend became clearer for both PBMCs (T2: p = 0.1782, T3: p = 0.0012) and granulocytes (T2: p = 0.0045, T3: p = 0.0005). Similarly, FBM animals also showed higher whole blood O_2_^•−^ concentrations when combining data from both treatment groups (T2: p = 0.0426; T3: p = 0.0464). As visualized in **Figure 5A**, BMW data points were much more “consistent”, i.e., displayed a noticeably lower spread in O_2_^•−^ data than FBM animals. Interestingly, FBM animals also required much more cardiovascular support, which manifested in doubled median NoA infusion rates required to achieve hemodynamic targets during the experiment (FBM 1.76 μg/kg·min (IQR 0.8;2.3), BMW 0.86 μg/kg·min (IQR 0.56;0.98), p = 0.031).

As shown in **Figure 5B**, stimulated granulocytes from BMW animals had higher phagocytic activity than FBMs at T1 and T2, which had normalized by T3. Furthermore, the percentage of granulocytes engaging in phagocytosis gradually decreased in FBMs, while BMW cells increased the fraction from T2 to T3. Unstimulated granulocytes also differed regarding their oxidative PPP. In FBM granulocytes, the oxidative PPP flux Q3 decreased upon ASDH and HS, while it increased slightly in BMWs (**Figure 5C**). This relationship reversed from T2 to T3 for both strains, however, with overall marginal differences between groups. We further detected a higher increase in the TCA cycle flux F4 in BMW animals between measurement time points T2 and T3.

Interestingly, PBMCs showed comparable behavioral patterns in terms of strain-specific differences as granulocytes (**Figure 5D**). However, additionally to patterns in oxidative PPP and TCA cycle utilization, we saw that in FBM PBMCs, the pyruvate-derived fraction of acetyl-CoA (F2) decreased upon ASDH and HS while it increased in BMWs; mirroring the pattern of the oxidative PPP. This trend in F2 was exclusive to PBMCs.

### 3.3 A principal component analysis revealed systematic changes in immunometabolism due to ASDH and HS

Principal component analysis is a powerful tool to detect systematic patterns in larger datasets. It enables to detect links between parameters (in our case flux analysis, mitochondrial respiration and O_2_^•−^ production) by describing the variation of the data with fewer dimensions, called principal components. Data from all three measurement time points were included to find sets of fluxes/parameters that behave in systematic manners in response to ASDH and HS. Here, the time frame from T1 to T2 should indicate metabolic adaptations induced by ASDH, HS and the intervention, while T2 to T3 represents effects of ongoing intensive care and, for the HyperOx group, the readaptation to normoxia.

As third components usually covered only a minimal set of fluxes with no recognizable pattern, we focused on the first and second component in our analysis unless mentioned otherwise. They are included in the component overview in **Table 1**, which describes PCA nomenclature (A-D), all components, their linked parameters, and their covered variance. In all performed PCAs that included all measurement time points, we could not find clustering of HyperOx vs NormOx, or FBM vs BMW groups. We therefore conclude that data variation was mainly caused by cell type and measurement time points and less by intervention or swine strain.

**Table 1:** Results from principal component analysis.

analyzed dataset	name PCA	#PC	var [%]	dir	metabolic pathways	linked parameters	separated groups
PBMCs & granulocytes all strains T1-T3	A	PC 1	36.8	+	glycolysis PPP	Glyc1, Glyc2, Q1, Q2, Q3, Q4, Q11, dTAL, F2, F5, Inp_Pyr_, L_ACC_, O_2_^•−^ production	granulo- cytes PBMCs **Figure 6A**
				−	TCA cycle	F3, F8, L_OAA_	
		PC 2	21.5	**+**		Inp_Pyr_	T1 → T3 **Figure 6A**
				−	TCA cycle	F3, F4, F6, F8, L_OAA_, L_Pyr_ Routine respiration	
		PC 3	11.5	+		F5, F9	
				−		F7	
PBMCs all strains T1-T3	B	PC 1	33.8	+	glycolysis non-ox PPP	Glyc1, Glyc2, Q1, Q2, Q11, dTAL, F4, F5, F7, L_Pyr_, Inp_Pyr_, L_ACC_, Routine respiration, O_2_^•−^ production	T1 → T2 **Figure 6B**
				−		L_OAA_	
		PC 2	21.9	+		Inp_Pyr_	T2 → T3 **Figure 6B**
				−	TCA cycle	F3, F4, F6, F7, F8, L_OAA_, Routine respiration, O_2_^•−^ production	
		PC 3	13.2	+		F2, F9, L_OAA_	
				−		F7	
granulocytes all strains T1-T3	C	PC 1	30.3	+	glycolysis PPP	Glyc1, Glyc2, Q1, Q2, Q3, Q4, Q11, dTAL, Inp_Pyr_	
				−		F6	
		PC 2	29.3	+		F2, L_ACC_	T1 → T3 **Figure 6C**
				−	TCA cycle	F3, F4, F6, F8, L_OAA_, Routine respiration	
		PC 3	10.0	+		F7	
				−			
granulocytes & stimulated granulocytes BMW T1-T3	D	PC 1	39.3	+	glycolysis non-ox PPP TCA cycle	Glyc1, Glyc2, Q1, Q2, Q11, dTAL, F3, F4, F5, F8, F9, L_OAA_, L_Pyr_	T1 → T3 **Figure 6D**
				−			
		PC 2	18.2	+	PPP	Q3, Q4, Q11, dTAL, L_ACC_, O_2_^•−^ production	stimulation **Figure 6D**
				−		F5	
		PC 3	17.6	+	non-ox PPP	Q11, dTAL	
				−		F7, L_ACC_	

We performed PCA with various datasets to identify hidden factors or processes of systematic metabolic change. Complete nomenclature of fluxes is provided in **Supplementary Figure 1**. var, variance; dir, direction.

**Figure 6:**
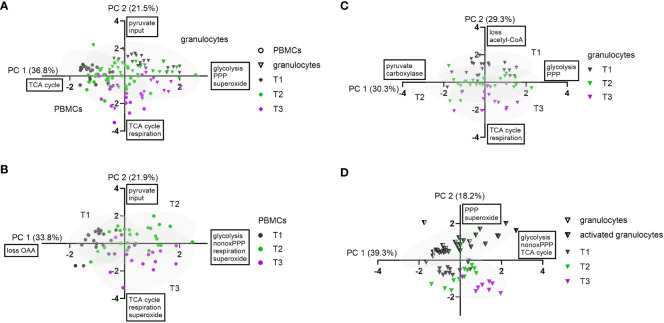
Principal component analysis of experimental data. Granulocytes are depicted as triangles and PBMCs as circles. Stimulated granulocytes are indicated as half-filled triangles. For the measurement time points, the following color code was chosen: T1: grey; T2: green; T3: violet. PC 1 and PC 2 scores were plotted in a x-y graph for each dataset. **(A)** Combined data from granulocytes and PBMCs (n = 123, FBM and BMW). Granulocyte and PBMC data are each depicted with their 95% confidence interval (CI) as grey ellipses. **(B)** PCA of combined PBMC data (n = 63, FBM and BMW). Data from each measurement time point are shown with their 95% CI. **(C)** PCA of combined granulocyte data (n = 60, FBM and BMW). Data from each measurement time point are shown with their 95% CI. **(D)** PCA of combined data of BMW granulocytes and *E.coli* bioparticle-stimulated granulocytes (n = 64, BMW). Data from granulocytes and stimulated granulocytes are each presented with their 95% CI.

PCA of all PBMC and granulocyte data revealed a cell type-specifc axis (A-PC 1, 37%) that confirmed increased O_2_^•−^ production, glycolysis, and PPP metabolism in granulocytes compared to PBMCs, while PBMCs displayed comparatively higher glutamine-fueled TCA cycle utilization. The latter comprised of higher glutamine uptake (F8), the TCA cycle flux F3 and oxaloacetate/aspartate loss (L_OAA_). A second axis matched negatively with progressing measurement time points (A-PC 2, 22%) and included fluxes of TCA cycle pathways and Routine respiration (**Figure 6A**).

When performing the PCA with only PBMC data, variation included two axes describing movement along measurement time points: B-PC 1 (34%) was positively linked to increased glycolysis, non-oxidative PPP (dTAL), Routine respiration and O_2_^•−^ production with values moving along this axis from T1 to T2. The second component (B-PC 2, 22%) was negatively linked with glutamine-fueled TCA cycle activity, Routine respiration and O_2_^•−^ production, implying that these pathways increased from T2 to T3 (**Figure 6B**). Even though there was no link between oxidative PPP activity and O_2_^•−^ production in either PC, Spearman correlations showed linear relations between superoxide production and the oxidative PPP flux Q3 (r< 0.57), TCA cycle flux F3 (r< 0.40), and Routine respiration (r< 0.52), respectively (data not shown).

In granulocyte data, a first component (C-PC 1, 30%) encompassed fluxes of increased glucose uptake (Q1), glycolysis and PPP. This axis could not be assigned to an identifiable data subset but made up a large proportion in data variance. A second component (C-PC 2) described increased glutamine-fueled TCA cycle fluxes including increased pyruvate carboxylase (F6), Routine respiration, and negatively linked pyruvate-derived acetyl-CoA (F2) and acetyl-CoA loss (L_ACC_). This component coincided with increasing measurement time points (**Figure 6C**). In contrast to PBMC data, there was no correlation between O_2_^•−^ production, TCA cycle or oxidative PPP activity (Q3). When including flux data from both the granulocyte and stimulated granulocyte dataset, we yielded one larger component linked to glycolytic, non-oxidative PPP (dTAL) and TCA cycle pathways (D-PC 1, 39%) and loosely aligning with increasing measurement time points, and a smaller second component (D-PC 2) explaining about 18% of data variance. The latter component unambiguously separated granulocytes from stimulated granulocytes and entailed variance in PPP fluxes and O_2_^•−^ production (**Figure 6D**). The third, comparatively large component entailed further variation in non-oxidative PPP activity (D-PC 3, 18%).

## 4 Discussion

The present work investigated the effect of targeted hyperoxemia on alterations of immunometabolism in granulocytes and PBMCs in swine with (FBM) and without (BMW) cardiovascular comorbidity. Regarding the clinical parameters, both studies found improved modified Glasgow Coma Scale (MGCS) values in the hyperoxemia groups (7), but only FBM swine had yielded higher brain tissue PO_2_ levels during targeted hyperoxemia (8). Neither study reported any other major beneficial or deleterious effects of hyperoxemia during resuscitation and intensive care. As oxygen availability is a major factor in immunometabolism, our study intended to uncover potential undetected effects of targeted hyperoxemia, the swine strain used, and ASDH and HS on circulating immune cells. PBMCs and granulocytes were isolated from whole blood before ASDH and HS (T1), 24 h post ASDH and HS (T2), i.e. at the end of the exposure to targeted hyperoxia, and 48 h post ASDH and HS (T3). Effects of HyperOx or NormOx were expected to manifest at T2 earliest, as treatment was initiated only upon the beginning of resuscitation.

Neutrophilic granulocytes are severely impacted by substrate and oxygen availability (35). They comprise up to 50 − 70% of the leukocyte population, making them the most abundant circulating immune cells. Neutrophils fight pathogens with a variety of mechanisms including phagocytosis, respiratory burst, and neutrophil extracellular trap (NET) generation. The metabolism of neutrophils is known to be highly glycolytic. Metabolized glucose is broken down into two pyruvate molecules, of which the majority (~ 97%) is converted into lactate while ~ 2% is used to fuel minimal TCA cycle activity. Granulocytes contain less mitochondria than other circulating immune cells, but require intact mitochondrial function for maintaining extended neutrophil activity (36). The PPP, another glucose dependent pathway, is required for ROS production and NETosis (37, 38). This general behavioral pattern is represented in our data showing minimal mitochondrial respiration and otherwise heavy dependence on glycolytic pathways (A-PC 1). After incubation with *E.coli*-derived particles, we saw that both the O_2_^•−^ production and the oxidative and non-oxidative PPP activity were markedly increased (D-PC 2). Interestingly, phagocytic stimulation did not impact lactate production. Awasthi et al. have referred lactate production as measured by extracellular acidification to be essential for NETosis (39). Hence, our data suggests a lactate-independent focus on respiratory burst and phagocytosis rather than NETosis by porcine neutrophils that were stimulated *ex vivo*.

With progressing measurement time points, granulocytes increasingly engaged in glutamine-fueled TCA cycle utilization, correlating with increased mitochondrial respiration (C-PC 2). This metabolic adaptation can be explained by greater fractions of immature granulocytes with experiment progression (due to HS and/or blood sampling for immune cell isolation), as *de novo* synthesized granulocytes typically rely on TCA cycle activity and oxidative phosphorylation during differentiation (40–42). The increase in TCA cycle activity was linked with aspartate production (aspartate/oxaloacetate “loss”), which is essential for nucleotide and protein synthesis. The linkage of respiration in this axis can be explained by proliferating cells requiring respiration for aspartate synthesis (43, 44). However, while Sullivan et al. and Birsoy et al. attributed the increase in aspartate synthesis to reductive carboxylation of α-ketoglutarate, our model could not confirm these assumptions, as the utilization of this pathway was not reflected in our labeling patterns (e.g. a higher ^13^C_5_ glutamine tracer-induced m+3 fraction on aspartate). A second axis detected by PCA encompassed glycolysis and both oxidative and non-oxidative PPP activity including ribose-5-phosphate synthesis, indicating variance in neutrophil activation and proliferation (C-PC 1). Surprisingly, in contrast to activated granulocytes, there was no relation or linkage between O_2_^•−^ production and oxidative PPP utilization in unstimulated granulocytes. This suggests that baseline O_2_^•−^ production in granulocytes was supported by pathways other than the PPP.

Investigation of PBMCs was more complex than granulocytes due to the very diverse nature of this cell population. Interestingly, by applying PCA we saw that they moved along two distinct metabolic axes over the course of the experiment: During ASDH, HS and subsequent resuscitation, PBMCs firstly increased glycolysis and non-oxidative PPP activity (B-PC 1), and later TCA cycle activity including glutamine uptake and oxaloacetate/aspartate production during long term intensive care (B-PC 2). Both axes were linked with increased mitochondrial respiration and O_2_^•−^ production. The linkage of TCA cycle activity, aspartate production, and Routine respiration of the later measurement time points has been similarly observed in granulocytes, while the correlation of O_2_^•−^ production with TCA cycle activity, Routine respiration and non-oxidative PPP activity was exclusive to PBMCs. Even though we saw a correlation between O_2_^•−^ production and the oxidative PPP flux Q3 according to the Spearman test, neither component included a linkage of these two parameters. This raises the possibility that changes in O_2_^•−^ production might have been mediated by superoxide formation within the respiratory chain (45). Li et al. suggested mitochondrial electron transport chain-derived O_2_^•−^ as a source of ROS in monocytes and macrophages, which might contribute to this relation (46). Interestingly, Zink et al. reported no effect of ASDH on PBMC metabolism using the same methods, although with a much more rudimentary metabolic model (47). Moreover, while ASDH was combined with HS in our current study, Zink et al. had investigated ASDH alone in the absence of HS and, hence, without systemic oxygen debt. Therefore, the PCs 1 and 2 aligning with time-dependent effects might be mainly attributed to HS as opposed to ASDH.

The FBM swine strain is characterized by a homozygous R84C mutation in the low-density lipoprotein receptor, leading to a genetic predisposition for atherosclerosis and coronary artery disease whose progression was promoted by a cholesterol-enriched diet. Hypercholesterolemia is known to promote inflammation, e.g., *via* neutrophil activation accompanied by NETosis and elevated ROS production (48). We saw that FBM animals presented with both elevated O_2_^•−^ concentration levels in whole blood and O_2_^•−^ production rates by granulocytes (T2: p = 0.0045; T3: p = 0.0005) and PBMCs (T3: p = 0.0012). In conclusion, the FBM phenotype increased O_2_^•−^ concentration in whole blood and O_2_^•−^ production rates by immune cells after induction of ASDH and HS, with a more delayed impact on PBMCs. As mentioned before, O_2_^•−^ production as measured with ESR was not linked to fluxes of the oxidative PPP and, in conclusion, acts independently in non-stimulated cells, while phagocytic activation leads to a mutual increase. As a metabolism- and phenotype-independent cause we suspected an impact of noradrenalin infusion, since FBM animals required more NoA support during ASDH and HS than BMW animals. However, the role of catecholamines, in particular NoA, on immune cell function in shock and sepsis is complex and cannot be easily mapped (49). There are a variety of known pro- and anti-inflammatory mechanisms, like pro-inflammatory activity *via* activation of α-receptors (50, 51), high doses of NoA leading to increased ROS and apoptosis in PBMCs (52), prevention of immune cell mobilization in murine trauma (53), or potential deactivation of catecholamines by auto-oxidation (54). Therefore, an atherosclerosis-induced effect on O_2_^•−^ production that is not mediated by the metabolic pathways covered in this study has higher plausibility. Another potential factor could be the age of animals, as FBMs were about twice as old as BWM animals due to the required feeding period of the cholesterol-enriched diet (a median of 38 *vs* 16 months, respectively). Overall, FBM and BMW were overlapping in the PCA and could therefore not cleanly be separated based on different flux behavior in ^13^C-MFA analysis.

While the effect of hypoxia on circulating immune cells is often a topic of investigation, information on hyperoxia-induced changes on metabolism is scarce. Our results suggest that targeted hyperoxemia had attenuated the granulocyte-specific increase in glutamine-fueled TCA cycle activity 24 h post shock. Sagone et al. suggested impaired mitochondrial activity in lymphocytes by hyperoxia, although our results did not replicate the effect of hyperoxia on *i)* glycolysis, *ii)* mitochondrial respiration, and *iii)* other lymphocyte subsets (55). Nevertheless, our study suggests a specific inhibition of TCA cycle acceleration in circulating granulocytes in response to ASDH and HS, without affecting mitochondrial respiration. This could also be caused by a decreased fraction of immature granulocytes. In contrast to granulocytes, there was no consistent effect of hyperoxia across multiple analyzes in PBMCs, except for a slight trend in the utilization of lipid synthesis indicated by the NADPH-providing oxidative PPP and the acetyl-CoA loss. Cells impacted by targeted hyperoxia had a more pronounced decrease in their lipid biosynthesis from the end of differential treatment until the end of the experiment than cells from the control group. However, this effect was only present in animals without cardiovascular co-morbidity.

### 4.1 Limitations of the study

As mentioned above, the diversity of PBMCs greatly complicated data interpretation. Metabolic plasticity could both be attributed to metabolic switches and changes in population composition. To a lower degree, similar problems arose for granulocytes in terms of mature/immature subsets with different metabolic profiles. Another limitation was sample availability: especially for the NormOx FBM granulocyte T1 data set there were problems with limited cell numbers after purification, as cell requirements were high but the maximally tolerable amount of blood drawn at the individual measurement time points had yielded too few granulocyte numbers. Missing data points of early experiment terminations exacerbated this issue. This was especially relevant for principal component analysis, which greatly benefits from rich data sets, and for statistical analyzes with repeated measures. A third limitation was caused by flux analysis, as not all flux determinations were equally precise. Most reliable were TCA cycle and glycolytic fluxes, while fluxes with greater distance from metabolites captured with GC/MS suffered in precision. This was especially the case for “small” inputs and outputs at pyruvate and acetyl-CoA nodes and for PPP fluxes, as the latter could only be determined through lactate labels. Main PPP metabolites, i.e., sugar phosphates, could not be captured with our derivatization method.

## 5 Conclusion

In a model of resuscitated porcine ASDH and HS, we saw that ventilation with increased inspiratory O_2_ concentrations to achieve target a P_a_O_2_ = 200 – 250 mmHg for 24 h after treatment initiation did not impact mitochondrial respiration of PBMCs or granulocytes. However, Bayesian ^13^C-MFA results indicated a reduction in TCA cycle activity in granulocytes compared to cells exposed to normoxemia in the same time period. This change in metabolism did not seem to affect granulocytes’ ability to perform phagocytosis or produce superoxide radicals. Animals with a hypercholesterolemic phenotype presented with higher superoxide burden than healthy pigs.

## Data availability statement

The raw data supporting the conclusions of this article will be made available by the authors, without undue reservation.

## Ethics statement

The animal study was approved by Regierungspräsidium Tübingen, Germany, Reg.-Nr. 1316. The study was conducted in accordance with the local legislation and institutional requirements.

## Author contributions

EW: Formal analysis, Investigation, Methodology, Software, Writing – original draft, Writing – review & editing. JV: Conceptualization, Formal analysis, Methodology, Resources, Software, Supervision, Validation, Writing – original draft, Writing – review & editing. MH: Investigation, Methodology, Software, Writing – review & editing. UW: Investigation, Methodology. NS: Investigation. TK: Investigation, Validation. TD: Investigation, Validation. DM: Investigation, Writing – review & editing. AH: Investigation. MG: Investigation. FM: Investigation. RM: Investigation. SM: Investigation. TM: Funding acquisition, Project administration. PA: Investigation, Project administration. EC: Conceptualization, Investigation, Methodology. PR: Writing – review & editing, Conceptualization, Funding acquisition, Project administration, Resources, Supervision, Writing – original draft. FZ: Conceptualization, Formal analysis, Investigation, Methodology, Writing – original draft, Writing – review & editing.
