# Supplementary material for: 13C-Metabolic flux analysis detected a hyperoxemia-induced reduction of tricarboxylic acid cycle metabolism in granulocytes during two models of porcine acute subdural hematoma and hemorrhagic shock
Source: Front Immunol. 2024 Jan 9;14:1319986. doi: 10.3389/fimmu.2023.1319986 (PMC10850868; doi:10.3389/fimmu.2023.1319986)
Supplement: Supplementary file 1 [file DataSheet_1.pdf]

frontiers

*Supplementary Material*

# $^{13}C$ Metabolic flux analysis detected a hyperoxemia-induced reduction of tricarboxylic acid cycle metabolism in granulocytes during two models of porcine acute subdural hematoma and hemorrhagic shock

**Eva-Maria Wolfschmitt[1*], Josef Albert Vogt[1], Melanie Hogg[1], Ulrich Wachter[1], Nicole Stadler[1], Thomas Kapapa[2], Thomas Datzmann[3], David Alexander Christian Messerer[1,4], Andrea Hoffmann[1], Michael Gröger[1], Franziska Münz[1,3], René Mathieu[5], Simon Mayer[5], Tamara Merz[1,3], Pierre Asfar[6], Enrico Calzia[1], Peter Radermacher[1], Fabian Zink[1]**

*** Correspondence:** Eva-Maria Wolfschmitt: eva-maria.wolfschmitt@uni-ulm.de

## 1 Metabolic modeling in RStan

We have established a combined model for the core metabolic processes glycolysis, pentose phosphate pathway (PPP), and the tricarboxylic acid (TCA) cycle. The model was implemented in RStan (R interface to Stan), a library for Bayesian modeling that utilizes user-defined models and data to return posterior simulations of prior defined parameters (1). To find posterior distributions of our unknown parameters (e.g. fluxes), random values are drawn for the parameter set from a prior defined range. From this set, predictions for data are calculated. In our case, these predictions are theoretical mass distributions for metabolites, which are then compared to the actual gas chromatography/mass spectrometry (GC/MS) measurement data. If the difference between predictions and measurements falls within the bounds of the expected measurement error, the parameter set are considered “true” values of the underlying distribution and collected in a Markov chain Monte Carlo (MCMC) sampling chain. This chain of “true” samples is used to calculate posterior mean, standard deviation, and credibility intervals. We worked with mass isotopomer distributions (MIDs) and not positional labeling. We therefore only considered the amount of labeling on the whole fragment and not the exact position of the isotopic $^{13}C$ label. Furthermore, in contrast to most approaches by other groups our definition of $^{13}C$ label includes the natural $^{13}C$ abundance, while all other natural isotopic variances were corrected for. We have made all corrected carbon mass distributions (CMDs) and $^{13}CO_2$ productions required for model calculations available in the supplementary tables.

The theoretical model used for calculations is visualized in Figure S1, with abbreviations being assigned in Table S3 and the list below. Each node represents a metabolite with its isotope labeling and each arrow a flux contributing to the metabolite mass distribution of the respective node. Their input-output flux balances are presented in Table S3. The calculation of theoretical mass distribution, e.g. through condensation of two metabolites, was based on the elementary metabolite unit (EMU) approach (2–5). To optimize for speed and to ensure efficient calculation, we reduced the model to a set of essential labeling patterns (oxaloacetate, acetyl-CoA), while the labeling on other metabolites were derived from the latter (6). We utilized multiple models: The first calculated ratios from CMDs, the second lactate production rates from measurements of the supernatant, and a third transformed ratios and production rates into absolute flux values. Parameter bounds and priors are listed in Table S5 (first model) and S6 (third model). For more detail about equations for EMU levels and modeling strategies, we refer to the already published supplement of Wolfschmitt et al. (7).

**Abbreviations and notations:**

- αKG: α-ketoglutarate pool
- ACC: acetyl-CoA pool
- CMD: carbon mass distribution
- $F_n$: flux within the TCA cycle
- $Glyc_n$: glycolytic fluxes
- Inp: input
- $L_n$: loss of metabolite due to compartmentalization
- MID: mass isotopomer distribution
- OAA: oxaloacetate pool
- $Q_n$: flux within the PPP
- $R_n$: flux ratio within the TCA cycle
- $S_n$: flux ratio within the PPP
- t: tracer input

# 2 Supplementary Figures and Tables

## 2.1 Supplementary Tables

**Table S1:** Components of mitochondrial respiration medium MiR05.

| | amount | material |
|---|---|---|
| D-sucrose [mM] | 110 | Sigma Aldrich, St. Louis, MO, USA |
| K-lactobionate [mM] | 60 | Sigma Aldrich, St. Louis, MO, USA |
| Ethylene glycol tetra acetic acid [mM] | 0.5 | Sigma Aldrich, St. Louis, MO, USA |
| Bovine serum albumin free from essential fatty acids [g/L] | 1.0 | Sigma Aldrich, St. Louis, MO, USA |
| $MgCl_2$ [mM] | 3.0 | Scharlau, Hamburg, Germany |
| taurine [mM] | 20 | Sigma Aldrich, St. Louis, MO, USA |
| $KH_2PO_4$ [mM] | 10 | Merck, Darmstadt, Germany |
| HEPES [mM] | 20 | Sigma Aldrich, St. Louis, MO, USA |

**Table S2:** End concentrations of important components in the three different RPMI media.

| | **1,2-$^{13}C_2$ glucose medium** | **$^{13}C_6$ glucose medium** | **$^{13}C_5$ glutamine medium** |
|---|---|---|---|
| unlabeled glucose [g/L] | 0.9 | 0.9 | 2.0 |
| labeled glucose [g/L] | 0.9 | 0.9 | - |
| unlabeled glutamine [g/L] | 0.6 | 0.6 | - |
| labeled glutamine[g/L] | - | - | 0.6 |
| $NaHCO_3$ [g/L] | 0.1 | 0.1 | 0.1 |
| HEPES [g/L] | - | - | - |
| RPMI stock (Sigma Aldrich, St. Louis, MO, USA) | RPMI-1640 R1383 | | RPMI-1640 R1145 + 1 mg/L folic acid |

**Table S3:** Input-Output relations at different metabolite pools.

| **Symbol** | **Metabolite** | **Input** | | **Output** |
|---|---|---|---|---|
| ***a*** | *ACC* | $F_2 + F_7$ | = | $F_4 + L_{ACC}$ |
| ***o*** | *OAA* | $F_3 + F_6 + F_9$ | = | $F_5 + F_4 + L_{OAA}$ |
| ***akg*** | *aKG* | $F_4 + F_8$ | = | $F_3$ |
| ***ci*** | *citrate* | $F_4$ | = | $F_4$ |
| ***suc*** | *succinate* | $F_3$ | = | $F_3$ |
| ***pyr*** | *pyruvate* | $Glyc_1 + F_5 + Inp_{Pyr}$ | = | $Glyc_2 + F_2 + F_6 + L_{Pyr}$ |
| ***h*** | *hexose* | $Q_1 + Q_5 + Q_9$ | = | $Q_2 + Q_3 + Q_6 + Q_{10}$ |
| ***p*** | *pentose* | $Q_3 + Q_6 + 2Q_8$ | = | $Q_4 + Q_5 + 2Q_7$ |
| ***e*** | *erythrose* | $Q_6 + Q_9$ | = | $Q_5 + Q_{10}$ |
| ***s*** | *sedoheptulose* | $Q_7 + Q_{10}$ | = | $Q_8 + Q_9$ |
| ***tr*** | *triose* | $Q_5 + Q_7 + Q_{10}$ | = | $Q_6 + Q_8 + Q_9 + Q_{11}$ |

**Table S4:** List of TBDMS derivatives of metabolite fragments as measured with GC/MS (8).

| Metabolite | carbon | m/z |
|---|---|---|
| ***glutamate*** | $c_1$ - $c_5$ | 432 – 439 |
| ***glutamate*** | $c_2$ - $c_5$ | 330 – 336 |
| ***aspartate*** | $c_1$ - $c_4$ | 418 – 423 |
| ***aspartate*** | $c_2$ - $c_4$ | 390 – 395 |
| ***aspartate*** | $c_1$ - $c_2$ | 302 – 308 |
| ***lactate*** | $c_1$ - $c_3$ | 260 – 264 |
| ***lactate*** | $c_2$ - $c_3$ | 232 – 236 |

**Table S5:** Important primary model parameters and their priors. Lowercase letters indicate transformed parameters. stdPrior = 0.3

| parameter | parameter bounds | model prior | transformation |
|---|---|---|---|
| ***$r_1$*** | <lower=−7,upper=7> | ~ normal(ln(0.9), 8*stdPrior) | ln(x/(1−x)) |
| ***$r_2$*** | <lower=−7,upper=0> | ~ normal(ln(0.84), 3*stdPrior) | ln(x) |
| ***$r_3$*** | <lower=−7,upper=0> | ~ normal(ln(0.7), 3*stdPrior) | ln(x) |
| ***$r_4$*** | <lower=−7,upper=0> | ~ normal(ln(0.5), 3*stdPrior) | ln(x) |
| ***$r_5$*** | <lower=−7,upper=0> | ~ normal(ln(0.03), 3*stdPrior) | ln(x) |
| ***$r_6$*** | <lower=−7,upper=0> | ~ normal(ln(0.02), 3*stdPrior) | ln(x) |
| ***v*** | <upper=0.0> | ~ normal(−0.41, 3*stdPrior) | ln(x) |
| ***k*** | <upper=0.0> | ~ normal(−0.51, 3*stdPrior) | ln(x) |
| ***w*** | <upper=0.0> | ~ normal(−0.41, 3*stdPrior) | ln(x) |
| ***z*** | <lower=−7,upper=7> | ~ normal(0, 6*stdPrior) | ln(x/(1−x)) |

**Table S6:** Important tertiary model parameters and their priors.

| parameter | parameter bounds | model prior |
|---|---|---|
| ***$F_2$*** | <lower=0.025,upper=200> | ~ normal(5, 4) |
| ***$F_3$*** | <lower=0.025,upper=200> | ~ normal(5, 4) |
| ***$F_4$*** | <lower=0.025,upper=200> | ~ normal(5, 4) |
| ***$F_5$*** | <lower=0.025,upper=200> | ~ normal(5, 4) |
| ***$Q_4$*** | <lower=0.025,upper=200> | ~ normal(15, 15) |
| ***$F_{11}$*** | <lower=0.025,upper=200> | ~ normal(10, 10) |
| ***$L_{OAA}$*** | <lower=0.025,upper=200> | ~ normal(3, 1.5) |

## 2.2 Supplementary Figures

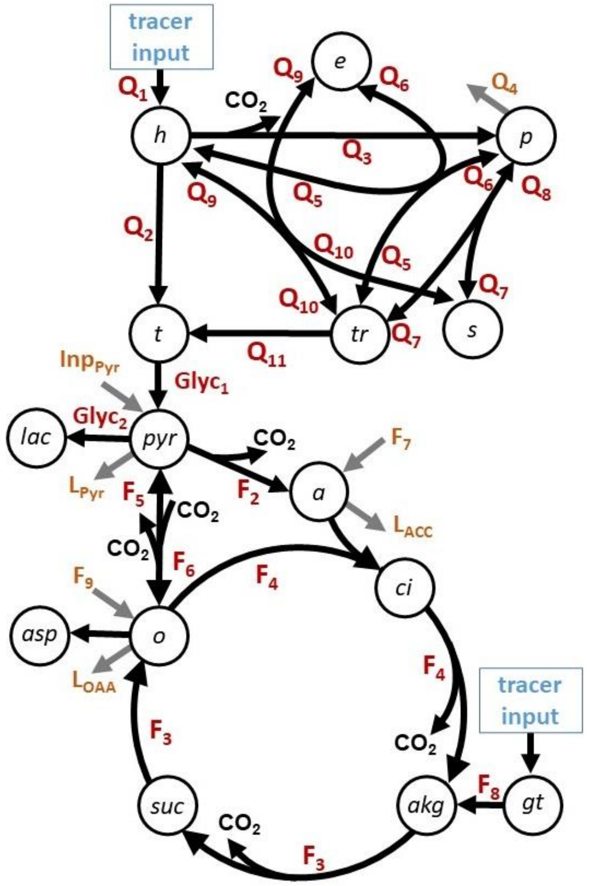


**Figure S1.** Visualization of the model used for Bayesian sampling. Glyc: glycolytic flux. Q: flux within the PPP. F: flux within the TCA cycle. L: flux leaving the network. Abbreviations are explained in Table S3.

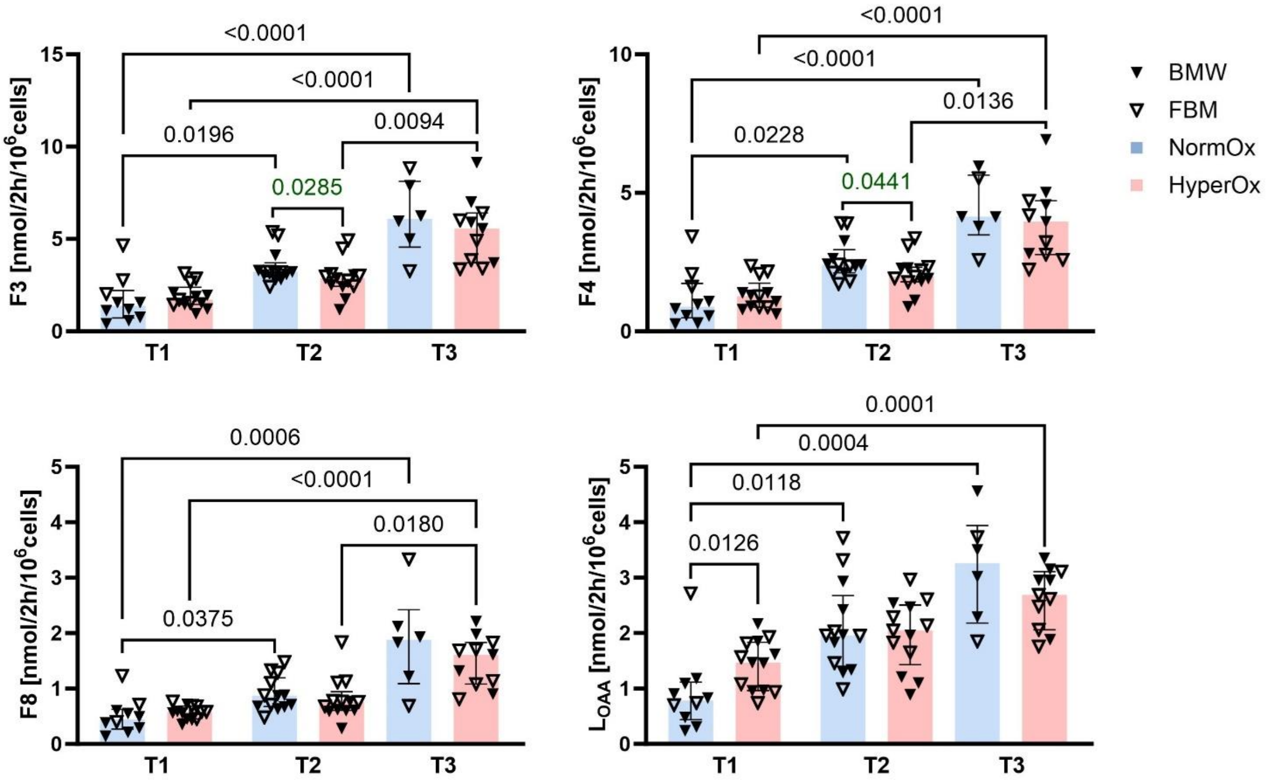


**Figure S2. Effects of targeted hyperoxia on circulating granulocytes.** Filled symbols indicate data from BMW animals, empty symbols FBM data. NormOx data are shown as blue bars, HyperOx as red bars. Absolute flux values of TCA cycle fluxes in isolated granulocytes including all measurement time points. F3: flux from $\alpha$-ketoglutarate to oxaloacetate, F4: oxaloacetate to $\alpha$-ketoglutarate. F8: glutamate to $\alpha$-ketoglutarate, $L_{OAA}$: oxaloacetate/aspartate leaving the modeled network. P-values are indicated in the graphs; Mann-Whitney U test for intergroup differences, Kruskal-Wallis rank sum test for time-related effects. Data are presented as median with IQR.

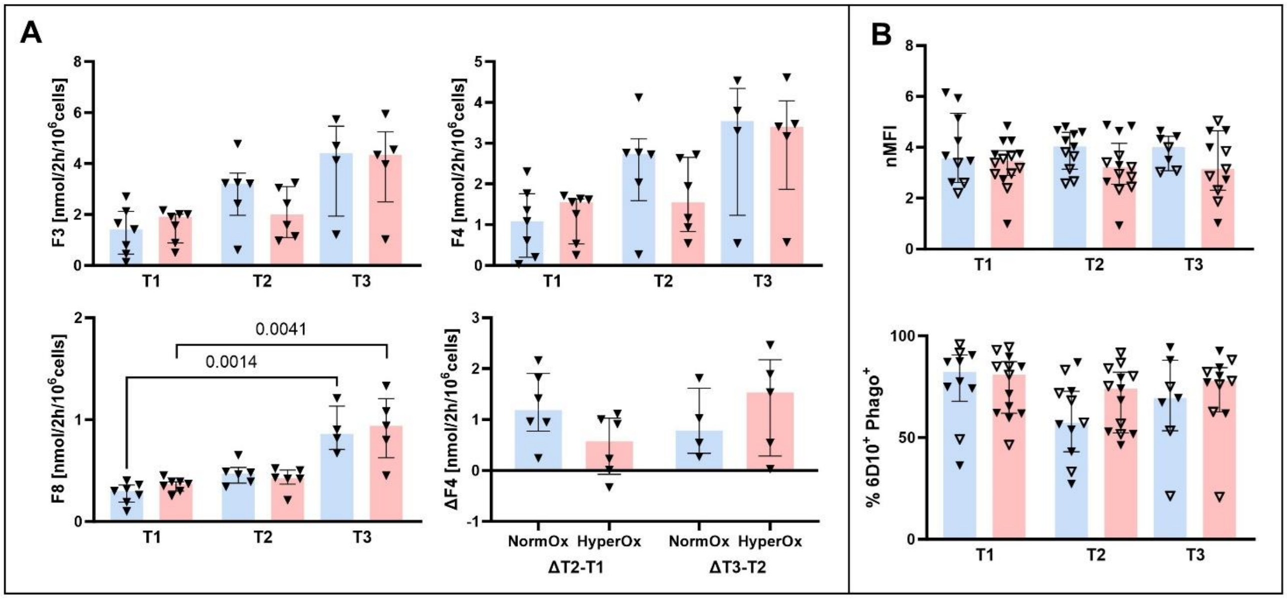


**Figure S4. Effects of targeted hyperoxia on *E.coli* bioparticle-stimulated granulocytes.** Filled symbols indicate data from BMW animals, empty symbols FBM data. NormOx data are shown as blue bars, HyperOx as red bars. Data are presented as median with IQR. **A)** Time-related behaviour of fluxes in HyperOx and NormOx groups in isolated granulocytes after stimulation. Data are either presented as absolute flux values or as the difference between measurement time points. F3: flux from $\alpha$-ketoglutarate to oxaloacetate, F4: oxaloacetate to $\alpha$-ketoglutarate. F8: glutamate to $\alpha$-ketoglutarate. **B)** Phagocytic activity of *E.coli* bioparticle-stimulated granulocytes. Data is either presented as mean fluorescence intensity (nMFI) or fraction of phagocytic granulocytes within the granulocyte subset (6D10$^{+}$ Phago$^{+}$). P-values are indicated in the graphs; Mann-Whitney U test for intergroup differences, Kruskal-Wallis rank sum test for time-related effects.

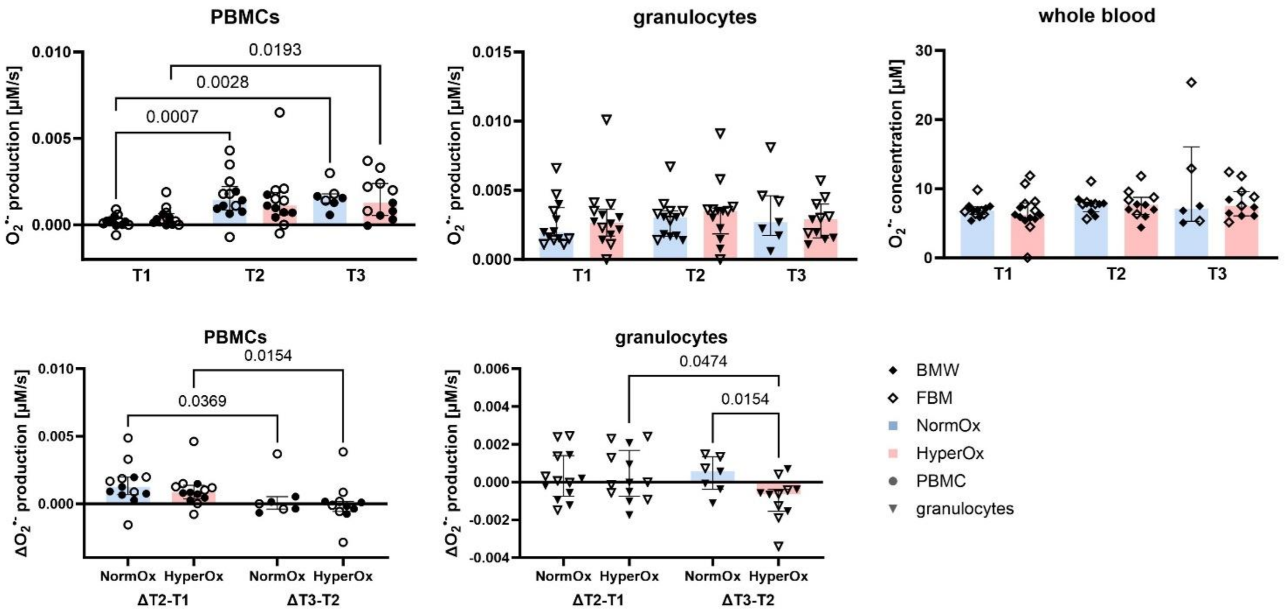


**Figure S4: Effect of targeted hyperoxia on $O_2^{\bullet-}$ production.** Filled symbols indicate data from BMW animals, empty symbols FBM data. NormOx data are shown as blue bars, HyperOx as red bars. $O_2^{\bullet-}$ production by PBMCs (circles) and granulocytes (triangles) and $O_2^{\bullet-}$ concentration in whole blood (diamonds) as determined by ESR at the indicated measurement time points. Data are either presented as absolute flux values or as the difference between measurement time points. P-values are indicated in the graphs; Mann-Whitney U test for intergroup differences, Kruskal-Wallis rank sum test for time-related effects. Data are presented as median with IQR.

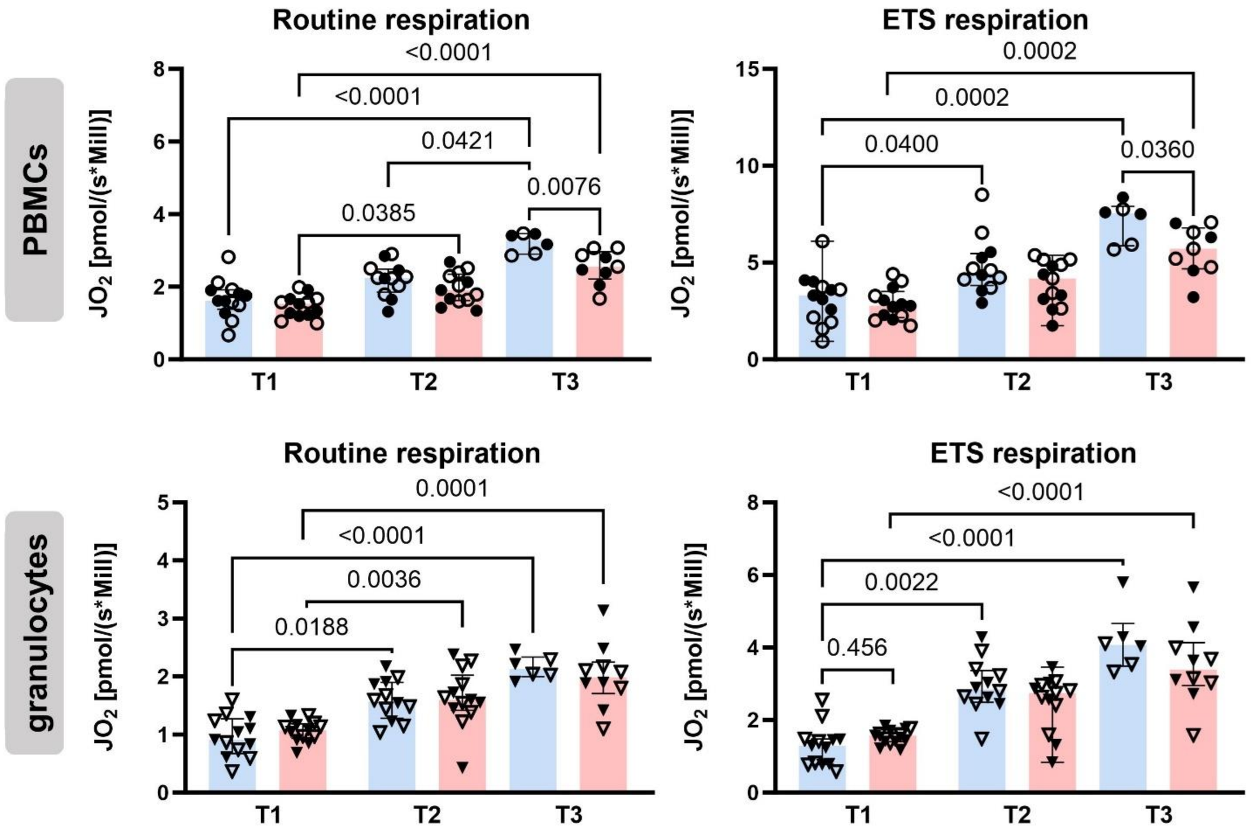


**Figure S5. Effects of targeted hyperoxia on the mitochondrial respiration of circulating immune cells**. Respiration was measured as Routine and ETS in PBMCs (circles) and granulocytes (triangles). Filled symbols indicate data originating from BMW animals, empty symbols FBM data. NormOx data are shown as blue bars, HyperOx as red bars. P-values are indicated in the graphs; Mann-Whitney U test for intergroup differences, Kruskal-Wallis rank sum test for time-related effects. Data are presented as median with IQR.

## 3 REFERENCES


1. Stan Development Team. RStan: the R interface to Stan (2020). Available from: http://mc-stan.org/
2. Weitzel M, Nöh K, Dalman T, Niedenführ S, Stute B, Wiechert W. 13CFLUX2--high-performance software suite for (13)C-metabolic flux analysis. *Bioinformatics* (2013) **29**:143–5. doi:10.1093/bioinformatics/bts646
3. Alger JR, Sherry AD, Malloy CR. tcaSIM: A Simulation Program for Optimal Design of 13C Tracer Experiments for Analysis of Metabolic Flux by NMR and Mass Spectroscopy. *Curr Metabolomics* (2018) **6**:176–87. doi:10.2174/2213235X07666181219115856
4. Antoniewicz MR, Kelleher JK, Stephanopoulos G. Elementary metabolite units (EMU): a novel framework for modeling isotopic distributions. *Metab Eng* (2007) **9**:68–86. doi:10.1016/j.ymben.2006.09.001
5. Wiechert W. 13C metabolic flux analysis. *Metab Eng* (2001) **3**:195–206. doi:10.1006/mben.2001.0187.
6. Vogt JA, Yarmush DM, Yu YM, Zupke C, Fischman AJ, Tompkins RG, et al. TCA cycle flux estimates from NMR- and GC-MS-determined 13Cglutamate isotopomers in liver. *Am J Physiol* (1997) **272**:C2049-62. doi:10.1152/ajpcell.1997.272.6.C2049
7. Wolfschmitt E-M, Hogg M, Vogt JA, Zink F, Wachter U, Hezel F, et al. The effect of sodium thiosulfate on immune cell metabolism during porcine hemorrhage and resuscitation. *Front Immunol* (2023) **14**:1125594. doi:10.3389/fimmu.2023.1125594
8. Antoniewicz MR, Kelleher JK, Stephanopoulos G. Accurate assessment of amino acid mass isotopomer distributions for metabolic flux analysis. *Anal Chem* (2007) **79**:7554–9. doi:10.1021/ac0708893
